# Dynamics of particle transport from soils to the sea

**DOI:** 10.1098/rsos.242159

**Published:** 2025-06-11

**Authors:** Don E. Canfield, Amin Naemi

**Affiliations:** ^1^ Department of Biology, University of Southern Denmark, Odense, Denmark

**Keywords:** river, weathering, soils, marine, sediment, trace metal, suspended sediment, bottom sediment

## Abstract

In this study, we explore the fate of soils, from their erosion into rivers to their final deposition as either continental or marine deposits. We focus on the continental United States and compare the chemistries of river suspended and bottom sediment with the chemistry of the surface soils from which these particles originate. We find that river bottom sediment is closer to the chemical composition of soils than to suspended sediment, implying that a large fraction of surface soils end up as river bottom sediment. We identify Zr/Al as a robust tool to differentiate river suspended and bottom sediment, and we use this tool to calculate that in the rivers studied, *ca* 60% of weathered rock mass ends as river bottom sediments and *ca* 40% is transported as suspended load. The Zr/Al ratio of marine sediments is close to the ratio in river suspended sediments, and we calculate that marine sediments comprise greater than 90% river suspended material. Overall, through the pre-Anthropogenic Holocene, approximately 50% of the particles eroded from the soils of continental United States accumulate in continental deposits, with the rest being transported to the sea. The principles outlined here could prove useful in exploring the dynamics of soil transport to the sea in the geologic past.

## 1. Introduction

Through continuous weathering and erosion, dissolved and particulate materials are cycled from land to the oceans, forming sediments and sedimentary rocks that are returned to the continents by various mechanisms. Combined, these processes are known as the rock cycle [1,2], and this cycle maintains the long-term regulation of Earth climate, ocean chemistry, as well as the continuous availability of elements to sustain life [2–5].

The weathering process begins as weathering fluids interact with rocks at the Earth surface. This interaction chemically alters the rock yielding mineral products, typically clays, with chemical compositions that differ from the parent rock, while liberating solutes to surface waters [2,6,7]. In addition to forming new mineralogies, the chemical weathering process also liberates relatively refractory minerals from the rock matrix, and in the end, weathering produces soils composed of particles of various sizes and with a chemical composition that may differ substantially from the parent rock [7–9].

The weathering process is also sensitive to climate, where variables like temperature and run-off can impact the intensity of weathering, element mobility and thus the chemistry of weathered particulates as represented, for example, by river suspended and bottom sediments [10–14]. Thus, the so-called ‘mobile elements’ like Na, K, Mg, Ca, Si, Rb, Sr, Cs and Ba [15,16] are typically more highly mobilized from rock to solution under conditions of higher rainfall and/or higher temperature and are therefore often depleted in river particulates compared with expected crustal average values [10–14,17].

Overall, the chemical archives of the weathering process are weathered soils, the suspended and river bottom sediments derived from the erosion of soils, and the marine sediments collecting these particles. However, the pathways leading from soils to rivers, and finally to marine sediments, are often complex, and this complexity may impact the chemistry of particles along this transport path. Thus, the pathway from soils to rivers begins with initial stages of erosion followed by the downslope transport and redeposition of eroded particles [18,19]. There may be size-sorting during these processes. For example, when soils are eroded by splash erosion, rainfall can disrupt soil aggregates and dislodge fine particles, preferentially liberating them from the soil matrix for further transport by overland flow towards streams and rivers [19–24]. In addition, water flowing through soil pore space in unsaturated soils can mobilize and remove the fine particles through a matrix of larger particles [25,26]. Therefore, coarser particles can be eroded from soils at different times than fine particles, where, for example, coarse particles may be mobilized during large storm events by overland flow, and especially as water becomes channelled into rills and gulleys with high flow velocities. After mobilization, coarse particles may be redeposited downslope in response to reduced transport velocities as rain events wane and/or as the slope of the transport system becomes reduced. Thus, the pathway from soil to rivers is complex, and fine particles transported to rivers may have a different history than the coarser particles.

When finally transported into rivers, particles may become further size differentiated, as for example, with the deposition of coarser materials into alluvial and channel deposits as river slope and water velocity decrease downstream. Furthermore, particles are transported in rivers as either suspended sediment or bedload depending on their grain size and local hydrodynamics [27,28]. The suspended fraction generally consists of smaller particles, while the bedload consists of larger, denser, particles [29–32]. However, some bedload can become mobilized during high flow and some suspended sediment can resediment under low flow. Overall, the timescales for river sediment transport can vary from days to millennia, with suspended material transport typically reflecting shorter timescales than the bedload [33]. Both the variable timescales of river particle transport and the additional size sorting of particles in rivers can lead to further complications in tracking the pathways and processes of particle delivery from soils to the sea. All in all, different particle histories, and the impact of hydrodynamics on particle sorting, can contribute to important chemical differences between suspended and bedload sediment [29–32].

In recognition of the multiple sources and pathways of particle transport from soils to rivers, there has been much work to understand river sediment provenance (see reviews in e.g. [34,35]). These studies use a variety of sediment properties to track sediment sources including fallout radionuclides, sediment mineralogy, sediment magnetic properties and a variety of geochemical and isotopic signatures. Such studies have proved very successful in elucidating, for example, the contribution of different sediment source types, often differentiated into land-use types (e.g. woodlands, channel bank, pasture, cultivated), erosion style (channel erosion, surface erosion) or geological provenance, as they contribute to the river suspended or bottom sediment load [29,30,34,36–39]. Such studies will typically measure several geochemical tracers in potential source regions, and in river suspended or bottom sediment loads, and will usually focus on specific size range of particles (e.g. less than 63 μm) to help mitigate against size-sorting effects. With this approach, studies use statistical methods to isolate which of the full range of tracers measured can best differentiate specific source regions and thus account for river sediment chemistry [34,35].

Thus, it is well known that particles sourced from different soil types and erosional styles can impact river particle chemistry with further chemical differentiation into the suspended and bottom sediment loads. However, most studies are regional in approach. Indeed, we are aware of only one other study exploring on a continental scale how soils contribute to the chemistry of the river suspended load [40]. While quite informative, this study does not consider chemical partitioning into the bottom sediment load nor how, quantitatively, soils are ultimately transported to the ocean.

We aim to address these issues here, where we characterize the similarities and differences between river bottom and suspended sediment from several major drainage basins of the continental United States. We then compare these chemistries with the chemistry of soils supplying particles to the basins. From these analyses, we evaluate how soils are quantitatively partitioned during erosion into the suspended and bottom load of rivers. We also identify key chemical differences in river suspended and bottom sediment, allowing us to evaluate the role of both suspended and bottom sediment in supplying particles to marine sediments. We find that the Zr/Al ratio is the most robust chemical indicator differentiating river suspended and bottom sediment. Thus, together with the new chemical analysis of 122 different marine sediments from around the globe, and literature data, we quantitatively assess the importance of both river suspended and bottom sediment in supplying particles to the ocean. This approach could represent a tool for exploring river particle dynamics in Earth’s past.

## 2. Methods

Suspended sediments were collected from May to June 1986, except for the Connecticut River, which was sampled in April 1987 [10]. River bottom sediment was collected as submerged sediment from near the suspended sediment sampling site. In many cases (11 rivers), duplicate samples of bottom sediment were taken. Suspended sediment samples were filtered onto either 0.4 μm Nucleopore (preferred) or Millipore 0.45 μm filters (for rivers with high suspended sediment loads) as described earlier [10]. Samples were allowed to air dry naturally. While major element concentrations in the suspended load were previously determined in [10], they are redetermined here to assure consistency with the methods used, as we also determine the composition of river bottom sediment.

We also analysed a total of 122 marine sediments from our collection and from the core collection of the Lamont-Doherty Earth Observatory. For the marine sediments, samples (500 mg) were suspended in 50 ml distilled water and gently mixed for several minutes. The samples were then filtered to remove pore water constituents (salt) dissolved into the distilled water. This procedure was repeated, and the filtered particles were dried at 40°C before further analysis. For elemental analysis, all river and marine sediment samples were well homogenized, and a portion of the dried sample (*ca* 100 mg) was weighed, in duplicate or triplicate, into cleaned Teflon^®^ bombs. The samples were then microwave digested in a solution of HNO_3_ : HF : HCl in a ratio of 3 : 2 : 1 (3 ml total) for 10 min at 180°C after a 15 min ramp to the digestion temperature. This procedure ensured effective reaction between HF and the rock and mineral matrices. A triplicate set of blanks were digested similarly, but without any added sample. Also digested were certified marine sediment standard MESS4 and stream sediment NCSDC 73309. In addition, elemental yields were monitored by spiking some sediment samples with a standard mixture of both high and low element concentrations containing all the elements we analysed.

After digestion, 10 ml of 4% boric acid solution was added to neutralize the acidic solution, to complex the Si released during the digestion as well as the fluoride. The boric acid thus improves Si recovery and inhibits the precipitation of fluoride minerals. After this, the Teflon^®^ vessels were reclosed and digested in the microwave for 30 min at 170°C after a 15 min ramp to the digestion temperature. This second microwave treatment aids in completing the complexation reactions and in redissolving fluoride minerals precipitated during the initial microwave treatment. Samples were then cooled and diluted both 20 and 400 times for inductively coupled plasma mass spectrometry (ICP-MS) analysis. A typical ICP-MS analytical run included standards of 0.1, 0.5, 1, 10, 100, 500 and 5000 μg l^−1^ together with milli-Q purified water as a blank. Standards and blanks were matrix equivalent with the samples. Recovery of standard samples was typically between 90 and 110%, while recovery of the elemental spikes was typically 100 ± 5%. Relative s.d. values of samples run in triplicate were generally within 2–3%, but were up to 32% for Cd, and occasionally 10–12% for some of the other elements. All recovery and s.d. results are shown in the electronic supplementary material. From our marine sediment analyses, we report only Al and Zr concentrations, where spike recovery was close to 100% for Al, with a relative s.d. of 1–2% for triplicate analyses. For Zr, spike recovery was between 90% and 115%, with a relative s.d. of 1–4% for triplicate analyses.

We explore a variety of parameters including basin lithology and soil chemistry in the context of the hydrologic unit (HU) occupied by the river. HUs are defined by the United States Geological Survey [41] and are classified at four levels. Thus, the first level is the largest geographical representation and includes one or a series of whole river basins, while the fourth level is the smallest and includes part or all of an individual drainage basin, or a combination of drainage basins defining a hydrologic feature [41]. Generally, we chose the largest HU to define the river upstream of the sampling site, but in some cases, such as the Mississippi and Missouri Rivers, for example, we chose to evaluate the HUs of intermediate size, and more proximal to the sampling site rather than evaluating the whole of the river basin. This choice reflects the probability that for large river systems, the chemistry of river sediment at a given location, and in particular, bottom sediment chemistry, is likely to be impacted by input from local sources [42]. Furthermore, for the Ohio, Mississippi, Missouri and Platte Rivers, sediment yield increases dramatically downstream [43], and it is high in the regions of our downstream samples. This observation implies that the more downstream sources contribute substantially to the sediment dynamics of these rivers.

We summarized the lithology of the rocks within the different basins from which our rivers drained. Lithologic information was taken from the US Geological Survey (USGS) geologic map of the United States [44], and we summarized the lithologies within the HU representing our different river basins. We also extracted data from the US soil database [45,46]. This database represents 4857 sites in the conterminous US that were analysed for major and minor element chemistry. Analyses were performed in the top 0−5 cm depth, representing the A horizon (uppermost mineral soil), the B horizon, representing the depth of intermediate weathering intensity and the C horizon, representing, in principle, partially weathered parent material. We have chosen here to concentrate on the A-horizon, as this horizon probably contributes to most particles liberated from soils during run-off and erosion. We also note that the chemistries of the A and B horizons are typically quite similar [45–47]. Soil data were partitioned into the HU defining each drainage basin as described above. The soil data within a given HU was used to compare with river particulate chemistry.

## 3. Results and discussion

### 3.1. River basins

**Figure 1.**
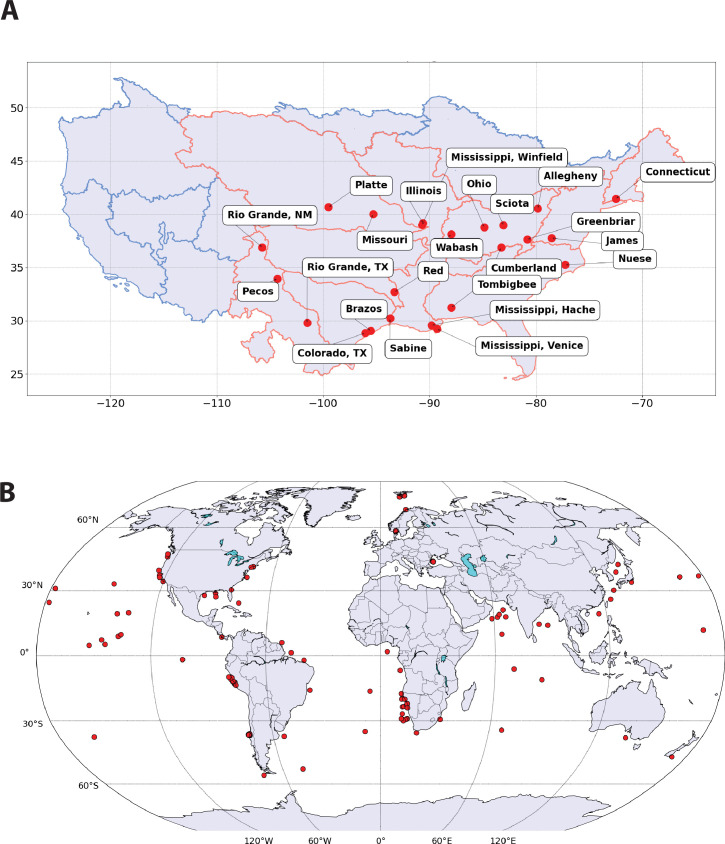
(A) Sampling location for river suspended and bottom sediment particulates together with an outline of the major (first level) HUs defining the river basins (see text) of the present study. HUs outlined in red are part of the drainage basins represented by river samples from the current study, whereas HUs outlined in blue are not. (B) Sample location for marine sediment particulates included in the present study.

The location of the rivers sampled, together with an outline of the first level HUs housing each river basin, are shown in figure 1 (digital masks for higher-level HUs are not available). The major characteristics of the river basins explored here are summarized in table 1, along with the specific HUs that we assigned to the individual rivers. The river basins of the present study drain approximately two-thirds of the continental United States and most of the area east of the Continental Divide, although individual river drain basins are of vastly different size (table 1). The basins represent a wide range of climate with seasonal average temperature varying between 4.4°C and 21°C and run-off varying between 0.83 and 59 cm y^−1^ (table 1). Sedimentary clastics, limestones and alluvium dominate the lithologies drained by the rivers studied here, although crystalline rocks are prominent in some of the drainage basins, particularly those draining mountainous terrains (table 1).

**Table 1.** Sampling location, physical characteristics, drainage basin designation (HU) and major drainage basin lithology.^a^

river	lat	long	basin area km^2^ x 10^3^	discharge m^3^ s^–1^	temp ºC	run-off cm y^–1^	HU^b^	%clay	%silt	%sand	lithology
Greenbriar	37.65	−80.81	3.5	55.4	10	49	50500	43.1	45.8	11.1	limestone, shale
Brazos	29 057	−95.535	89.2	179	18.9	6.3	1207	71.3	27	1.7	alluvium, sandstone, shale
Colorado, TX	28 845	−96.063	80.6	64.3	20	2.5	1209	88.5	16.2	1.3	sed clastics, volcanic, unconsolidated
Connecticut	41 443	−72.462	25.1	457	7.2	57	108	24.9	73.5	1.6	basalt, shale, schist, gneiss, sandstone
Cumberland	36 882	−83.262	4.2	74.4	12.8	56	513	61.3	34	4.7	limestone, shale
Illinois	39.16	−90.614	67.7	616	11.7	29	713	62.4	36.6	1	limestone, shale
Red	31 265	−91.959	133	496	17.8	11.8	1114	49	47	4	alluvium, sed clastics
Mississippi, Winfield	38 996	−90.68	309	1780	8.9	18	7	70.8	28.2	1	limestone, shale
Mississippi, Venice	29 239	−89.305	2970	16200	21	17.2	8	31.7	59.1	9.2	alluvium
Missouri	40 018	−95.307	1150	1150	7.2	3.1	1024	36.4	60.8	2.8	sed clastics, alluvium, granite, metabasalt
Neuse	35.26	−77.232	7	79.1	17.2	36	302	64.8	34.2	1	unconsolidated
Ohio	38 773	−84.844	216	3300	10	48	509	ND	ND	ND	sed clastic, limestone
Pecos	33 944	−104.302	29.6	20.4	12.2	2.2	1306	35.9	47.4	16.7	lava, alluvial
Platte	40 677	−99.493	146	48.6	7.8	1.1	1020	40.6	50.7	8.7	sed clastics, uncosolidated
Rio Grande, TX	29 784	−101.512	210	55.4	15.5	0.83	13	60.2	38.9	0.9	alluvium, unconsolidated, limestone, granite
Rio Grande, NM	36 898	−105.731	17.7	20.9	5.6	3.7	1302	30.4	65.2	4.4	granit, schist, gneiss, alluvium
Sabine	30 223	−93.712	19.4	139	20	23	1201	ND	ND	ND	alluvium, unconsolidated
Sciota	38 963	−83.043	10	95.7	11.7	30	506	ND	ND	ND	limestone sed clastics
Tombigbee	31 234	−87.964	40	645	17.2	51	316	48.6	49.3	2.1	alluvium, limestone, sed clastics
Wabash	38 131	−87.942	3402	332	11.7	30	512	48.7	50.3	1	limestone, shale
Allegheny	40 535	−79.812	23.3	438	7.2	59	50 100	38.8	59.4	1.8	sed clastics
James	37 763	−78.518	11.9	143	13.9	38	208	35.2	61.5	3.3	unconsolidated, granite, shale

^a^ Basin area, average discharge, air temperature, run-off and %clay, %silt, %sand of the suspended fraction taken from [10]

^b^ Hydrologic Unit

### 3.2. Element data presentation and pollution

The major element and minor element data for the suspended sediments are presented in electronic supplementary material, table S1 together with total organic carbon (TOC) and inorganic carbon (IC) data from [10]. Major element data (Mg, Fe, Mn, Al, Ca, Na, K and Si) were also presented in an earlier publication [10], and the new data generated here agree with the previously published data to generally within 20% or better. The major and minor element data for the bottom sediment are shown in electronic supplementary material, table S2. Duplicate samples of bottom sediments were usually very close in composition (within ±5–10%), except for the elements Te, Mn, Ta, Cd, W, Tl, Th and U, where differences were as large as a factor or 2−3, but only in some cases. The river sediment data presented here are available through Mendeley at: https://data.mendeley.com/datasets/63b8fy55jc/1.

Pollution can potentially impact our river data and thus its interpretation, as sources of pollution (industry for example) may not originate from soils. Pollution will be an issue mostly for metals with industrial applications such as Zn, Cu, Ni, Cr, Mo, Cd, Sn, Sb, Ag and Pb. Indeed, in some of the rivers studied, the trace element concentrations of suspended sediment exceed by over a factor of 2 the highest concentrations observed in the other rivers studied (electronic supplementary material, table S1). This occurs for Cu, Ag and Sn in the Connecticut River, Cu, Zn, Cd and Sn in the Neuse River, and Ni, Cu, Zn in the Sciota River. While the high trace metal concentrations in these rivers could result from natural causes, pollution seems the most likely explanation. Indeed, elevated concentrations of Cd, Cu, Zn and Pb have been found in suspended sediments draining the Park River watershed, a tributary to the Connecticut River [48]. Likewise, sediment cores from the Neuse River estuary show a long history of trace metal contamination (Zn, Cu, Cd, Cr and Ni) dating back to 1925, although Ni and Cr enrichments are much reduced or absent from sediment deposited after *ca* 1980 [49]. We found no similar studies for the Sciota River, but it drains the highly industrial area of central Ohio. Therefore, in our calculations, we have removed from consideration the metals named above from the Connecticut, Neuse and Sciota Rivers.

### 3.3. Study limitations

Studies aimed at unravelling river sediment provenance often involve sampling river suspended and/or bottom sediment over a whole season and often at several depths in the river. When bottom sediments are collected, they are often obtained in several locations in the riverbed and possibly also over the year [29,30,34,35,38,50]. Our study is low resolution in that suspended sediment was collected only once, as close to river mid-depth as possible, while bottom sediment was also collected either once or twice (electronic supplementary material, table S2) near the site where suspended sediment was taken. Our low sampling density is clearly not ideal, but we think it is workable. For example, the bulk elemental compositions of suspended sediments collected over three seasons in the Ganges and Brahmaputra Rivers, including depth transects during the summer monsoon, showed a relatively low degree of variability, with s.d. from the mean in elemental concentration of typically 5–15% for most elements [30]. Also, from a seasonal study of rivers draining into the Great Lakes [51], it was concluded that single sampling of particle chemistry would very likely represent the particle chemistry transported by rivers into a lake. In contrast, river bottom sediment chemistry can be much more variable. For example, samples collected over distances of hundreds of kilometres in the Ganges River, and in different years, had variability with s.d.s of 6–30% for major elements, while variability in the trace elements was typically between 20% and 60% [29]. For the Brahmaputra River, variability was even higher, ranging between 15% and 60% for the major elements and between 6% and 150% for the minor elements [29].

We used the USGS soil database to estimate soil contributions to an individual river basin. This database was collected on a grid, and while soil samples were collected at different depths, they were not collected with special reference to land use types, erosional style or underlying geology, as is often done in provenance studies as noted above. Still, in a given river basin, multiple soil samples have been measured, although the variability in elemental composition between samples is typically high (electronic supplementary material, figure S1).

Finally, our marine database is rather large (figure 1), but the oceans are vast, and most regions are either unsampled or under-sampled with respect to element chemistry. Therefore, any conclusions we draw based on these data must be considered in the context of the available data. This consideration will be fully discussed below.

Despite these challenges and limitations, we believe that our dataset is sufficient to answer primary questions about the relationship between soils and the chemistry of river suspended and bottom sediments derived from them, as well as the particle source to marine sediments as developed and discussed below.

### 3.4. From soils to rivers

#### 3.4.1. Similarity of soils to river suspended and bottom sediments

Our first step in tracking the history of particles from soils to the sea is to explore the chemical relationship between soils of a river basin and their respective river bottom and suspended sediment loads. As noted above, numerous studies have explored this relationship using a variety of techniques, although these studies generally have more specific aims, such as, for example, deciding which land use types or erosion styles contribute to the suspended (typically) or bottom sediment load of rivers. As also described above, in conducting these studies, a wide variety of chemical constituents of different soils and river particles are typically analysed, and statistical methods are used to determine which of these best differentiates the particle sources. These specific chemical tracers are then used to source the particles in the river suspended or bottom sediment load. Our approach is more general, as we are interested in the broader relationship between soil chemistry and the chemistry of river suspended and bottom sediment, and across multiple river basins with continental-scale geographic coverage.

To make our assessment, we calculate a similarity index (SI) averaged across both the major elements and across all elements measured in our suspended and river particulates (minus the elements in those rivers viewed as impacted by pollution as noted above). We define the similarity index as


(3.1) $$SI=1-\left|\frac{C_{soil}-C_{river}}{max\left\{C_{soil},\;\;C_{river}\right\}}\right|,$$

where *c*_soil_ is the average concentration of a given element in the topsoil in the basin (the specific HU as listed in table 1), *c*_river_ is the concentration of the element in either the river suspended or bottom sediment load of the river draining the basin (the suspended and bottom sediment load are computed separately), and max {*c*_soil_, *c*_river_} is the highest value between the soil and river particle concentrations (either suspended or bottom sediment). Thus, with an SI of 1, soil and river particle concentrations are the same. With an SI of 0.80, particles with the lower concentration (either river particulates or soil) have concentrations 80% as high as those with the higher concentration, and with an SI of 0.5, particles with the lower concentration have concentrations 50% as high as those with the higher concentration. Overall, comparisons with higher SI values are more similar in concentration.

The SI values for river bottom and suspended particles are calculated for each element in each river basin and are presented in electronic supplementary material, table S4. Average SIs for all elements, and for the major elements (Na, K, Al, Mg, Ca and Ti), from each basin are presented in table 3 along with an average SI for all rivers. Note that the average SI values across all rivers are calculated from basin average soil, suspended and bottom sediment chemistries as presented in table 2, and not by averaging individual SI values for each of the rivers.

**Table 2.** Average values for river suspended sediment, bottom sediment and topsoil compared with crustal average values.

		crust ave	susp sed		bottom sed		soil	
			average	s.d.	average	s.d.	average	s.d.
Na	%	2.42	0.45	0.29	0.62	0.38	0.51	0.42
Mg	%	1.50	0.94	0.28	0.54	0.32	0.38	0.18
Al	%	8.15	8.32	1.29	4.41	1.56	3.82	1.22
Si	%	31.10	25.8	3.60	34.6	4.96		
K	%	2.32	1.92	0.48	1.58	0.50	1.24	0.52
Ca	%	2.57	2.34	3.26	1.96	2.54	1.43	1.79
Ti	%	0.38	0.44	0.08	0.37	0.25	0.26	0.07
Fe	%	3.52	4.77	1.15	2.14	0.84	1.83	0.53
Li	mg kg^−1^	24.0	41.5	15.5	22.7	10.2	19.70	6.63
Be	mg kg^−1^	2.10	1.83	0.37	1.12	0.41	1.21	0.37
P	mg kg^−1^	670	1390	1450	525	236	645	24
V	mg kg^−1^	97	112	19.68	53.8	21.7	49.7	14.2
Cr	mg kg^−1^	92	72.6	21.8	35.2	14.4	30.9	8.43
Mn	mg kg^−1^	770	1500	932	609	360	624	342
Co	mg kg^−1^	17.3	15.0	5.72	7.54	3.45	8.21	3.57
Ni	mg kg^−1^	47	50.2	40.0	16.1	8.3	13.78	5.92
Cu	mg kg^−1^	28	41.3	17.9	14.1	15.4	14.8	4.62
Zn	mg kg^−1^	67	190	82.6	61.2	32.5	62.0	22.8
Ga	mg kg^−1^	17.5	20.6	3.52	12.3	4.18	9.41	3.11
As	mg kg^−1^	4.8	12.1	2.84	6.10	2.96	6.17	2.41
Rb	mg kg^−1^	82	109	22.1	70.7	22.9	60.3	19.4
Sr	mg kg^−1^	320	171	103	177	150	112	83.2
Y	mg kg^−1^	21	25.4	3.54	18.4	5.82	13.7	4.03
Zr	mg kg^−1^	193	129	15.9	149	45.5		
Nb	mg kg^−1^	27	15.3	2.76	12.6	5.89	9.28	2.59
Mo	mg kg^−1^	1.1	1.89	1.38	1.00	0.89	1.22	0.89
Ag	mg kg^−1^	0.05	0.36	0.33	0.10	0.04		
Cd	mg kg^−1^	0.09	1.39	0.83	0.24	0.16	0.25	0.10
Sn	mg kg^−1^	2.10	6.57	5.13	2.06	1.32	1.61	0.67
Sb	mg kg^−1^	0.40	0.95	0.36	0.51	0.21	0.63	0.20
Cs	mg kg^−1^	4.90	6.81	1.01	2.84	1.40	5.25	0.41
Ba	mg kg^−1^	624	625	152	527	234	416	182
La	mg kg^−1^	31	39.3	6.50	25.1	7.82	25.7	6.48
Ce	mg kg^−1^	63	80.4	14.3	50.7	15.8	52.1	12.6
Pr	mg kg^−1^	7.1	9.32	1.66	6.16	1.89		
Nd	mg kg^−1^	27.0	36.3	6.63	24.3	7.50		
Sm	mg kg^−1^	4.70	6.95	1.30	4.65	1.47		
Eu	mg kg^−1^	1.00	1.37	0.24	0.93	0.29		
Gd	mg kg^−1^	4.00	5.81	1.06	4.05	1.24		
Tb	mg kg^−1^	0.70	0.78	0.14	0.58	0.18		
Dy	mg kg^−1^	3.90	4.89	0.77	3.55	1.07		
Ho	mg kg^−1^	0.83	0.90	0.13	0.67	0.20		
Er	mg kg^−1^	2.30	2.67	0.36	2.01	0.61		
Tm	mg kg^−1^	0.30	0.37	0.05	0.28	0.09		
Yb	mg kg^−1^	2.00	2.56	0.29	2.02	0.58		
Lu	mg kg^−1^	0.31	0.36	0.04	0.30	0.09		
Hf	mg kg^−1^	5.30	3.69	0.45	4.42	1.32		
Ta	mg kg^−1^	0.90	1.03	0.18	0.82	0.26		
W	mg kg^−1^	1.90	1.58	0.31	1.26	0.79	0.82	0.29
Tl	mg kg^−1^	0.90	0.87	0.20	0.49	0.27	0.43	0.16
Pb	mg kg^−1^	17.0	40.0	16.6	22.3	8.17	25.2	11.9
Bi	mg kg^−1^	0.16	0.42	0.14	0.14	0.07	0.19	0.07
Th	mg kg^−1^	10.5	11.6	1.75	7.39	2.07	7.79	1.82
U	mg kg^−1^	2.70	3.13	0.65	2.33	0.64	2.26	0.86

Values of SI calculated from the major elements are higher for the bottom sediments than for the suspended load, although not all the differences are significant (table 3). Likewise, when considering all the elements together, the average SI values for the bottom sediments are also higher than that for the suspended load, except for the Greenbriar River. For average SI values calculated across all rivers, values are much higher for bottom sediment than that for suspended sediment. Indeed, a bottom sediment SI of 0.78 for major elements shows that bottom sediments are 78% similar in composition to soils in average major element chemistry. An SI of 0.83 for all elements shows that when all elements are taken together, average river bottom sediments are 83% similar to average soil in chemical composition (table 3). For the suspended load, total element and major element particle chemistries are 53% and 57% similar, respectively, to average soil chemistry.

**Table 3.** Similarity index^a^ for suspended sediment (susp) and bottom sediment (bottom) compared with average soil for each river basin.

River	SI susp total	SI bottom total	SI susp Major^b^	SI bottom major
Greenbriar	0.64 (0.19)^c^	0.57 (0.19)	0.55 (0.22)	0.57 (0.19)
Brazos	0.48 (0.15)	0.65 (0.17)	0.46 (0.22)	0.49 (0.12)
Colorado, TX	0.60 (0.19)	0.82 (0.14)	0.55 (0.21)	0.86 (0.08)
Connecticut	0.39 (.18)	—	0.49 (0.22)	—
Cumberland	0.49 (0.22)	0.64 (0.16)	0.50 (0.24)	0.58 (0.15)
Illinois	0.66 (0.18)	0.83 (0.11)	0.60 (0.13)	0.88 (0.06)
Red	0.52 (0.21)	0.65 (0.15)	0.34 (0.22)	0.50 (0.22)
Mississippi, Winfield	0.50 (0.16)	—	0.54 (0.19)	—
Mississippi, Venice	0.53 (0.15)	0.60 (0.14)	0.53 (0.22)	0.56 (0.11)
Missouri	0.70 (0.16)	0.75 (0.19)	0.72 (0.14)	0.85 (0.09)
Neuse	0.32 (0.21)	0.67 (0.18)	0.40 (0.28)	0.68 (0.17)
Ohio	0.58 (0.19)	0.79 (0.12)	0.57 (0.14)	0.81 (0.09)
Pecos	0.56 (0.17)	0.80 (0.17)	0.58 (0.23)	0.75 (0.14)
Platte	0.62 (0.21)	0.77 (0.16)	0.59 (0.15)	0.84 (0.14)
Rio Grande, TX	0.71 (0.21)	0.81 (0.17)	0.66 (0.20)	0.84 (0.15)
Rio Grande, NM	0.61 (0.27)	0.77 (0.17)	0.72 (0.20)	0.76 (0.15)
Sabine	0.33 (0.14)	0.57 (0.26)	0.34 (0.16)	0.59 (0.20)
Sciota	0.60 (0.23)	0.72 (0.19)	0.59 (0.15)	0.69 (0.32)
Tombigbee	0.30 (0.14)	0.56 (0.21)	0.28 (0.14)	0.45 (0.15)
Wabash	0.61 (0.15)	0.71 (0.15)	0.56 (0.10)	0.65 (0.11)
James	—	0.62 (0.23)	—	0.48 (0.20)
All rivers average^d^	0.53 (0.15)	0.83 (0.11)	0.57 (0.17)	0.78 (0.07)

^a^ see equation (3.1)

^b^ major elements include: Na, Mg, Al, K, Ca, Ti and Fe

^c^ s.d.

^d^ Calculated from average elemental composition of the soils in the river basins represented by the study

The SI values can vary substantially between rivers. For example, considering total element chemistry, SI values for the suspended load are lowest (less than or equal to 0.5) for the Brazos, Connecticut, Cumberland, Mississippi, Sabine, Neuse and Tombigbee Rivers, and highest (greater than or equal to 0.6) for the Greenbriar, Colorado, Illinois, Missouri, Platte, Rio Grande (both Texas and New Mexico), Sciota and Wabash Rivers. There is also variability in the SI values for bottom sediment chemistry, where concentrating on total element chemistry, the lowest values (less than or equal to 0.6) are found for the Greenbriar, Mississippi (Venice), Sabine and Tombigbee Rivers, while the highest (greater than or equal to 0.8) are found in the Colorado (Texas), Illinois, Pecos and Rio Grande (Texas). We see no obvious explanations as to why the particle chemistries of some rivers deviate more from average soil chemistry than others. Indeed, the rivers with high and low SI values overlap in basin size, discharge rate and bedrock lithology (table 1). Indeed, it is possible that the rivers with particularly high SI values may be under-sampled with respect to either the representative soil chemistries or the average chemistries of river suspended and bottom sediment.

Still, we conclude that bottom river sediment is much closer to the average chemical composition of soils than the suspended load. This is true in both individual drainage basins and for average soil, suspended sediment and bottom sediment chemistries. This conclusion might seemingly conflict with studies showing how a mix of soils representing different land use types or erosional styles within a basin can nearly or fully account for the chemistry of the suspended sediment load [34,36–39]. As noted above, in these cases, a small selection of the measured chemical parameters fingerprint the different soil types. For example, in the Upper Torridge catchment in Devon, UK, (area 258 km^2^), samples were collected from soils representing four source types including: channel banks, areas under cultivation, pasture lands and woodlands [34], with 30–50 samples collected from each soil type. Samples were sieved to less than 63 μm and analysed for a total of 19 different chemical parameters including major and minor elements, N, C and the radionuclides: ^226^Ra, excess ^210^Pb and ^137^Cs. Of these, a combination of N, K, ^137^Cs, C, Fe and ^226^Ra, were found to fully distinguish the source types. Bulk riverine suspended sediment was also collected and filtered to less than 63 μm, and a multivariate mixing model was used to partition the relative contributions of the soil source to the suspended sediment load. Thus, the suspended load could be fully accounted for by this approach. Such an approach may be considered as typical in evaluating the source of river particulates [34,35].

How can one both successfully account for the river suspended load by mixing different soil sources, yet observe that soils are generally closer in composition to the bottom sediment than the suspended sediment load? This apparent contradiction is addressed by recognizing important differences between our approach and the approach outlined above in other studies. One difference is that the soil chemical parameters used to ‘fingerprint’ a soil type may not transport congruently with all soil chemical properties during erosion and transport. For example, as noted above, heavy minerals (zircon and rutile for example) may transport differently during erosion than less-dense clays and organic material leading to mineralogical and chemical separation during transport. In this way, some of the potential soil-fingerprinting elements may preferentially end with suspended particles, while others may preferentially end with sediment bed load.

Another difference is that studies on river sediment provenance often analyse only a specific size fraction of sediment, typically less than 63 μm. This is done to reduce the impact of large, unweathered, rock and mineral particles on chemical provenance assessment. We did not do this, and inclusion of such particles could explain some of the differences between our SI values for river bottom and suspended sediments, especially as large particles will concentrate in river bottom sediments.

#### 3.4.2. Quantifying suspended and bottom sediment loads

Some interesting questions arise from our conclusion that the chemical composition of soils better reflects river bottom sediment chemistry than the chemistry of river suspended sediment. One question is, if soils better reflect, and in some cases closely reflect, the chemical composition of river bottom sediments, where does the suspended sediment come from? The likely answer, as explored in §1, is that soils, for the most part, erode incongruently, where the fine-sized particles that dominate the suspended load are liberated preferentially from the soil matrix during processes like splash erosion and water flow through soil pore space. This winnowing of the fine particles leaves the soils more chemically similar to the river bottom sediments that form from the soils.

Since surface soils seem to end mostly as river bottom sediments, we can start from the perspective of unweathered surface materials and ask what is the relative production rate of suspended and river bottom sediment material during the weathering process? To answer this, we look for distinct chemical signals that can differentiate river bottom and suspended sediment. This is done by first calculating the enrichment factors for all elements analysed using equation (3.2) where ‘X’ is any element and ‘river’ is either average river suspended or bottom sediment value (table 2) and CA is the crustal average value [52],


(3.2) $${EF}_{x}=(X_{river}/{Al}_{river})/(X_{CA}/Al_{CA}).$$

**Figure 2.**
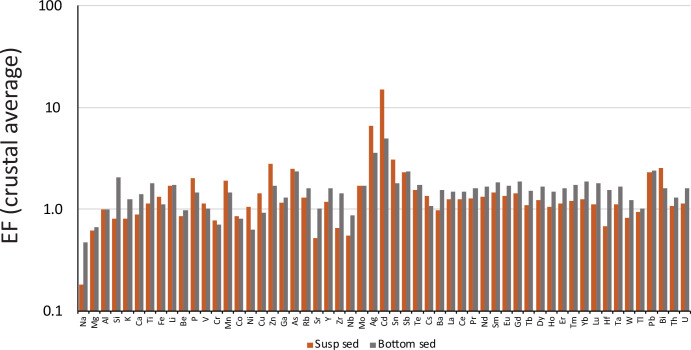
Enrichment factors (EF) of average suspended and bottom sediment of the present study relative to crustal average values. See text for details.

Results are shown in figure 2, and they show some large differences in EFs between river suspended and bottom particulates, especially for the elements Na, Si, Sr, Zr, Ag, Cd and Hf. Of these, the geochemical twins Zr and Hf are probably the most chemically conservative, not being impacted by diagenesis, authigenesis or other pathways of remobilization. These issues are of special importance when we take observations into marine sediments where numerous authigenic and diagenetic processes can impact the chemistry of the sediment. When considering between Hf and Zr, we focus on Zr, as measurements of Zr are generally more abundant than Hf (if we look beyond our own data) in literature accounts reporting both modern river particulate and marine sediment chemistry, as explored below.

Thus, we propose the Zr/Al ratio as a geochemical tool to fingerprint river bottom and river suspended sediment. If we think about their origins, Zr is mainly found in the heavy mineral zircon, and zircons can be weathered from the rock matrix and mobilized separately from aluminosilicates, allowing a different pathway of transport than other products of weathering like clays, for example. Being a heavy mineral, zircon can become concentrated in the denser bottom sediment fraction, explaining its elevated concentrations in this fraction. Furthermore, while Al can undergo post-depositional mineralogical alterations in marine sediments, like Zr, it is not diagenetically added to sediment. For these reasons, the Zr/Al ratio in marine sediments is a good source indicator. Indeed, the Zr/Al is often used as a geochemical provenance indicator. For example, Zr/Al has long been recognized as grain-sized proxy and tends to be elevated with aeolian dust input into marine sediments [53–57]. The Zr/Al has also been used to track the intensity of fluvial discharge as it impacts the particle source of marine sediments [58].

To develop this tool, we would ideally compare the Zr/Al ratio in bottom and suspended sediments with the same ratio in soils and in unweathered basement rock representing the basin from which the river particulates were derived. In doing so, we could encompass regional differences in the Zr/Al of unweathered source materials. Unfortunately, Zr was not measured in the US soil databases, and the bedrock analyses are too sparse [59] to be of use for most of the river basins studied. Therefore, we will analyse global composite averages of river suspended sediment and bottom sediment. Suspended sediment data come from the present study together with data from a previous compilation [60]. Likewise, the bottom sediment data are a combination of data from the present study and the compilation of [61]. In using the [61] compilation, only data from whole unmodified samples were used, as it is typical to sieve bottom sediment to a specific size fraction before analysis [17]. Size-fractionated samples, however, do not yield data comparable to our results. In addition, in using the dataset from [61], values for an individual river were averaged (data summarized in electronic supplementary material, table S5). The compilation of [61] also contains suspended sediment data, but the rivers with Zr and Al are mostly from New Zealand and are not representative of a global sampling. Therefore, we use the compilation of [60] to compliment our data for suspended sediment chemistry as it is much more globally representative [29].

**Figure 3.**
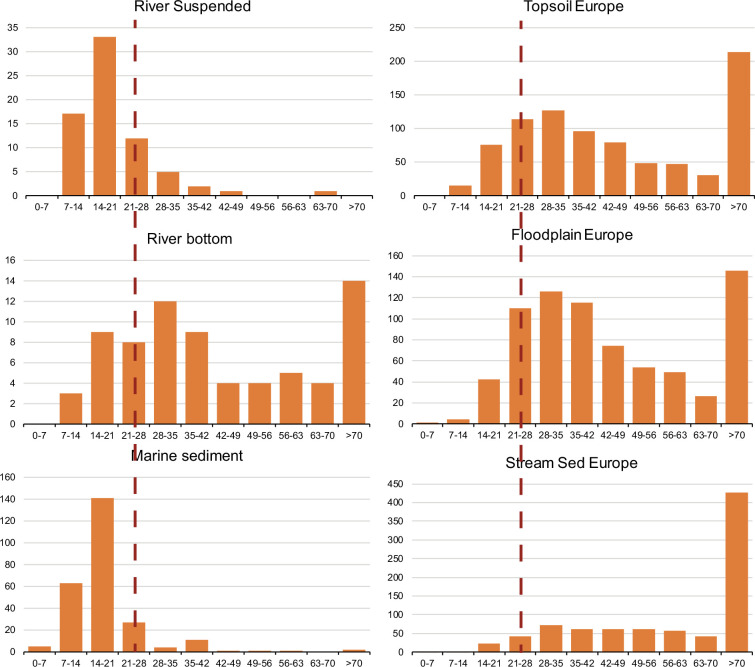
Frequency diagram of Zr/Al for river suspended and bottom sediment, marine sediments and topsoils, floodplain and stream sediments from Europe. The dotted red line is the crustal average value.

Overall, there is a clear distinction in the distribution of Zr/Al ratios between suspended and bottom sediment (figure 3). Furthermore, the peak Zr/Al ratio of the suspended sediment is well under the crustal average value of 23.7 [52], while the bottom sediment data generally exceed this value. These histograms confirm that Zr/Al can be a robust tool for differentiating river suspended and bottom sediment. Furthermore, as the maximum frequency distributions of Zr/Al for river suspended and bottom sediment straddle either side of the crustal average value, we propose that Zr and Al data can be used to estimate the relative production of suspended and bottom sediment during weathering. To do this, we assume, first, that unweathered surface material has a Zr/Al resembling the crustal average value at 23.7 [52]. Next, we assume that this surface material decomposes during weathering into particles with average Zr and Al concentrations resembling those of modern river suspended and bottom sediments. With these assumptions, we write the following mixing equation:


(3.3) $$CA=\frac{x\;\times \;Zr\;S+y\;\times \;Zr\;B}{x\;\times \;Al\;S+y\;\times \;Al\;B},$$

where CA is the crustal average value, *x* is the proportion of suspended sediment, *y* is the proportion of bottom sediment. Furthermore, ZrS is the average concentration of Zr in river suspended sediments, while AlS is the average Al concentration of river suspended material. Likewise, ZrB and AlB are the respective averages for river bottom sediment. With


(3.4) x+y=1

we solve for *y*,


(3.5) $$y=\frac{CA\;\times \;AlS-ZrS}{CA\;\times \;AlS-ZrS-CA\;\times \;AlB+ZrB}.$$

With the crustal average Zr/Al value, and the average Al and Zr concentrations for river bottom and suspended sediment (table 2), *y* = 0.63 and *x* = 0.37 (table 4). These results suggest that as a global average, more river bottom sediment is produced than suspended sediment during weathering.

**Table 4.** Input parameters and calculation results for the proportion of riverine bottom (*y*) and suspended sediments (*x*) formed during weathering (first three rows) and deposited into the sea (last row).

particle souce/fate	Zr/Al^a^	bottom sed		susp sed		prop bott	prop susp
	target	Zr ppm	Al wt%	Zr ppm	Al wt%	(*y*)	(*x*)
weathering	23.7	237	5.06	129	8.32	0.63	0.37
weathering	20	237	5.06	129	8.32	0.38	0.62
weathering	28	237	5.06	129	8.32	0.80	0.20
marine	16.1	237	5.06	129	8.32	0.03	0.97

^a^ For the calculations (using equation (3.5)) determining the proportion of bottom and suspended sediment produced during weathering (first three rows), the Zr/Al target is an estimation of the crustal average Zr/Al. The first row represents the true crustal average, while the next two assume values both greater than and less than the crustal average value (see text for details). For the last row labelled ‘marine’, the Zr/Al target is the average Zr/Al of marine sediments. From these calculations (equation 3.5), the value of *y* is determined, where *x* is 1*−y*.

There are, however, numerous uncertainties with this calculation including the assumption that unweathered surface materials have a crustal average composition. If we assume, instead that surface materials have an initial Zr/Al of 20, then the relative importance of suspended and bottom sediment reverse, with *y* = 0.38 and *x* = 0.62. In contrast, with an initial rock Zr/Al of 0.28, bottom sediments become even more important with *y* = 0.8 and *x* = 0.2. Another uncertainty relates to whether our suspended and bottom sediment analyses are truly representative of global river chemistries. For suspended sediment, we can only assume so, but for bottom sediment, we can compare bottom sediment chemistry with topsoils and floodplain sediments from Europe. Indeed, the European soils and floodplain sediments share a similar frequency distribution of Zr/Al values to our bottom sediment compilation (figure 3, data from FOREGS program; [62,63]), suggesting that such a frequency distribution may be common in soils and river bottom sediments. Curiously, stream bottom sediments from Europe also show a similar frequency distribution, but with a strong tail to very high Zr/Al ratios of greater than 60 (figure 3). Some of the high Zr/Al ratios in European soils are probably due to extensive aeolian deposits through central Europe [64], although the reason for the exceptional partioning of Zr into stream deposits versus those of river floodplains is not clear.

Overall, we conclude that rocks weather with a balance towards a higher production of bottom sediment. What, however, is the ultimate fate of these particles?

### 3.5. Particle transport to the sea

Rivers transport particles from soils, through terrestrial depocentres like river bottom and floodplain deposits, to the sea. We attempt to quantify the relative importance of river suspended versus river bottom sediment to marine deposits by comparing their respective Zr/Al ratios. The marine sediment samples from our compilation range in depth from 0.5 to 100 cm, but are mostly from the upper 15 cm, and probably represent deposition during last 500 years. As noted above, these data represent a broad global distribution (figure 1), and samples are taken from water depths ranging from 5 to 5300 m (electronic supplementary material, table S3). Overall, marine sediments have a very similar Zr/Al distribution to river suspended sediments (figure 3), suggesting that river suspended sediments are a primary source of particles to depositing marine sediments. Indeed, we can use equation (3.5) to calculate these relative contributions, replacing CA with the average Zr/Al of marine sediments at 16.1 (table 4). This calculation yields *y* = 0.03 and *x* = 0.97, confirming the suggestion that suspended sediments are the major source of marine particles. Our results are also broadly consistent with previous estimates on the sources of pre-human particle delivery to the oceans. These estimates include a suspended particle flux to the oceans of approximately 14 Pg y^−1^, with an additional *ca* 1−2 Pg y^−1^ delivered from river bottom sediment [65–67]. From these values we calculate that suspended sediments represent 86–93% of riverine particle delivery to the oceans, in line with our calculation that river bottom sediments contribute a relatively small proportion to marine sediments compared with the suspended load. In addition, there is an estimated 0.5–1.5 Pg y^−1^ contribution to marine sediments from aeolian sources [68,69] and 0.8 Pg y^−1^ from glacial melting [69] (table 5).

**Table 5.** Pre-Anthropogenic sources of particles to marine sediments.

particle source	flux
	Pg y^−1^
suspended sed	14
bottom sed	1−2
aeolian	0.5−1.5
glacial	0.8
suspended sed total	77–86%
other total	14–23%

References: [65–69].

Combined, non-riverine particle sources (e.g. aeolian and glacial sources) probably exceed the delivery of river bottom sediment to the oceans, but these particle sources are localized as to where they deposit. Glacial deposits largely impact Arctic regions, whereas aeolian sources, of which the Sahara desert is of particular importance [70–73], impacts preferentially the equatorial Atlantic Ocean and the Mediterranean Sea. The Zr/Al ratio of Saharan dust collected from atmospheric deposition has a spectrum of Zr/Al values that range widely [74] but are skewed towards higher values (electronic supplementary material, figure S2), quite different from the Zr/Al distribution of marine sediments from our compilation (figure 3). We are unaware of any Zr and Al analyses from glacial erosion, but we have probably under-sampled marine environments where both glacial and aeolian deposits are of particular importance. As these data become available, our analyses can be refined to capture the regional magnitude of these particle sources.

We note some additional caveats with our analysis. There are probably regional differences in climate/hydrology/topography that will impact the extent to which the suspended and bottom sediment loads will differ in their Zr/Al and, indeed, the extent to which the bottom load might contribute to the marine sediment deposition. For example, the sand and larger fraction of river bottom sediment from Taiwan [75] (electronic supplementary material, figure S2) show a Zr/Al distribution around the crustal average value, and values lower than average for global bottom sediments (figure 3). Indeed, a clustering around the crustal average for these bottom sediments could indicate that in environments with high relief and especially high erosion rates, such as Taiwan [67], there is little size differentiation of particle sizes into the suspended and bottom sediment fractions during erosion and transport. Thus, in these environments with high relief and high erosion rates, river particle transport to the oceans may be more efficient than in environments with lower relief. This aspect of particle transport to the oceans is missing in our analyses, but it could be incorporated with further analysis of river and marine sediments in areas of high relief.

Also, although our marine sediment dataset is well-spread geographically (figure 1), it may under-represent areas receiving particles from terrestrial environments of high relief and particularly high particle discharge such as the coast of Taiwan, as mentioned above. We also note that, in principle, to quantify the global importance of suspended and bottom sediment to the marine sediment pool using Zr and Al concentrations, data from individual marine sediments should be converted to burial fluxes by combining with sediment deposition rates. This would give a more accurate accounting of Zr and Al burial fluxes. We have not attempted this, though, as there is insufficient sediment deposition rate data associated with our marine sites. We note, however, that no significant trends in Zr/Al ratio are observed with water depth (electronic supplementary material, figure S3). As sedimentation rate roughly correlates with sediment depth [76], there would appear to be no inherent trends in Zr/Al with sediment deposition rates. Therefore, our assessment of global-scale marine sediment partitioning into suspended or bottom sediment from river sources may not be grossly compromised with the lack of detailed flux calculations.

### 3.6. Particle storage on land

From our analyses, we estimate that some 60% of the particles produced during continental weathering form river bottom sediments, and that only a small percentage (from 3% to 14% depending on estimate; see above) of these are transported to the sea with the remaining stored on land. These estimates, in principle, represent mass balances free of the impacts of modern dams and other man-made obstructions on particle transport to the sea [69], and are thus appropriate for the pre-Anthropogenic Holocene. Combining estimates for per cent bottom sediment production from soils and per cent bottom sediment transport to the sea, it is likely that greater than 50% of the particles liberated during weathering are retained on the continents. Indeed, hydrologists have known for decades that there is often a major imbalance between the rates of particle erosion and rates of particle transport by rivers, as expressed by the sediment delivery ratio (rate of sediment delivered at catchment outlet divided by gross erosion rate in basin [19,77,78]). Values of less than 1 represent particle storage in the river basin, and indeed, most river basins have sediment delivery ratios of less than 1. This ratio generally becomes smaller with increasing basin size to values of less than 0.1 in some basins [19,77]. The general trend of decreasing sediment delivery ratio with increasing basin size arises because larger rivers tend to experience larger overall decreases in channel slope through their course and more opportunities for particle storage in floodplains, bars and other geomorphic features [19]. Thus, large river basins tend to store a high percentage of the particles that enter the basin from erosion. There is, however, the expectation that over long-enough time periods, most or all particles derived from erosion will be delivered to sea, and that the delivery ratio should approach 1 for most basins [78]. This expectation may not be strictly true and could vary through time in the geologic past.

Independent assessments of particle depositional dynamics have been attempted for the Quaternary Period (2.58 Ma to present) based on the mass of preserved sediments on land and in the sea. These estimates suggest that over the Quaternary Period, continental clastic deposits have accumulated less mass (11.3–13.6 × 10^21^ g) than clastic marine sediments (24.7 × 10^21^ g) [79], amounting to approximately 30% of the total particle deposition. This rate of continental particle deposition does not wholly conform with our results, where we conclude that greater than 50% of the particles liberated by weathering during the pre-Anthropogenic Holocene deposited on the continents. However, the estimate of continental clastic deposition rates by [79] is based on poor constraints (according to [79]) on the volume of continental clastic deposits. Indeed, new geologic maps show that Quaternary-aged alluvial rocks and windblown loess cover approximately 18% of the land surface, and together, these represent the largest surface-exposed lithologic type [80]. These maps do not give sediment volume estimates, but they suggest that terrestrial river deposits have been important as sediment depocentres over the Quaternary. Still, it is also possible that both our estimate of continental particle storage, and that of [79], are equally valid, but relate to different timescales. The Quaternary experienced times of waning and waxing glaciation, and particle transport to the oceans might be more efficient during times of glacial melt than during the pre-Anthropogenic Holocene, the time period relevant for our analysis. We conclude, however, that particle storage on land over the last 2.58 Myr has been significant, accounting for up to, or even more than, 50% of the particle production from weathering during the pre-Anthropogenic Holocene.

## 4. Closing remarks

In the normal view of the rock cycle, the first step is the erosion of soils to the sea. This may be true over very long timescales, but on shorter timescales, the pathways from soil to the sea are complex. Indeed, soils erode into rivers contributing to both their bottom and suspended sediment loads. The chemical differences between the suspended and bottom loads are substantial, and soils, from which river particles derive, are chemically much more similar to the bottom load of rivers. This observation implies that a substantial proportion of the particles liberated during weathering end up as continental deposits. We recognize the Zr/Al ratio as a robust way to differentiate between river suspended and bottom sediment, and we use this chemical fingerprint to estimate the partitioning of particles into river bottom and suspended sediments during weathering and erosion. We also use the Zr/Al ratio to estimate the magnitude of river suspended and bottom sediment deposition into the ocean as well as to quantify particle storage on land during the pre-Anthropogenic Holocene. We find that *ca* 60% of the particles produced during weathering and erosion end up as river bottom sediment, while the remainder is transported as suspended sediment. With our data, 97% of the sediment depositing as marine sediments is derived from riverine suspended material. These values imply that greater than 50% of the particles produced during weathering are stored on the continents. This estimate is valid for the pre-Anthropogenic Holocene, and it is somewhat higher than an estimate of *ca* 30% particle storage for the Quaternary. On long timescales with changing climate and/or tectonic uplift of the continents, much of this continental alluvium may become eroded to the oceans. Exploring the Zr/Al ratio of marine sediments over time may provide a means to unravel the connection between soil erosion, continental storage and marine deposition through Earth history.

## Data Availability

All data is available in the online supplementary material uploaded with the manuscript [81].
